# Meniscus repair: up-to-date advances in stem cell-based therapy

**DOI:** 10.1186/s13287-022-02863-7

**Published:** 2022-05-16

**Authors:** Yixin Bian, Han Wang, Xiuli Zhao, Xisheng Weng

**Affiliations:** 1 Department of Orthopedic Surgery, State Key Laboratory of Complex Severe and Rare Diseases, Peking Union Medical College Hospital, Chinese Academy of Medical Science and Peking Union Medical College, Beijing, 100730 China; 2 Department of Medical Genetics, Institute of Basic Medical Sciences, Chinese Academy of Medical Sciences, School of Basic Medicine, Peking Union Medical College, Beijing, 100005 China

**Keywords:** Meniscus repair, Stem cell therapy, Regenerative medicine

## Abstract

The meniscus is a semilunar fibrocartilage between the tibia and femur that is essential for the structural and functional integrity of the keen joint. In addition to pain and knee joint dysfunction, meniscus injuries can also lead to degenerative changes of the knee joint such as osteoarthritis, which further affect patient productivity and quality of life. However, with intrinsic avascular property, the tearing meniscus tends to be nonunion and the augmentation of post-injury meniscus repair has long time been a challenge. Stem cell-based therapy with potent regenerative properties has recently attracted much attention in repairing meniscus injuries, among which mesenchymal stem cells were most explored for their easy availability, trilineage differentiation potential, and immunomodulatory properties. Here, we summarize the advances and achievements in stem cell-based therapy for meniscus repair in the last 5 years. We also highlight the obstacles before their successful clinical translation and propose some perspectives for stem cell-based therapy in meniscus repair.

## Introduction

The meniscus is a crescent-shaped fibrocartilage on the tibia articular surface. Normal meniscus deepens the depression of the tibial condyle and cushions the femur condyle, so as to enhance joint stability, facilitate joint lubrication, and maintain joint function [1–5]. As an essential component for the integrality of the knee joint, the meniscus bears a poor self-healing ability for its intrinsic avascular characteristics [1, 6–8]. Only the marginal 10–30% meniscus receives blood supply from the synovial membrane directly and can be healed after injuries, while the central meniscus nourished by the penetration of joint fluid lacks self-healing ability [7–10].

The annual incidence of meniscus injuries reaches 66–70 per 100,000 people, mainly caused by trauma and degenerative diseases [11–15]. Meniscus injuries lead to multiple clinical symptoms including joint pain, swelling, and locking. It’s estimated 50% of patients with persistent meniscus or anterior cruciate ligament tear will develop osteoarthritis or other articular cartilage degenerative diseases within 10–20 years [16] and the incidence of osteoarthritis will increase up to sevenfold for patients who went through meniscectomy [17]. Thus, meniscus injuries and related degenerative diseases propose a substantial burden to the healthcare system.

Various surgical therapies were applied to treat meniscus injuries including meniscectomy, allogeneic meniscus transplantation, and artificial meniscus implantation [18–24]. However, these therapies all bear some drawbacks. For instance, meniscectomy was reported to predispose the knee joint toward osteoarthritis and other degenerative changes [1, 14, 17]. The application of allogeneic meniscus was restricted by limited tissue availability, disease transmission risk, and mismatch between the graft and the host [21, 25, 26]. Concerning artificial meniscus, although the preliminary published data were promising, its long-term therapeutic effect is conflicting [27, 28]. Since no operative therapy for meniscus injury achieved satisfactory outcome currently, attentions were altered to alternative conservative strategies, among which stem cell-based therapy that possesses potent regenerative properties and can promote the natural healing process of the meniscus attracted a lot of interest [29–33].

As a regenerative strategy, stem cell-based therapy has achieved great advances in treating musculoskeletal diseases, such as bone and cartilage defects, osteonecrosis of the femoral head, and intervertebral disk degeneration disease [34–40]. For meniscus injuries that need reconstruct neo-cartilage, fibrous, and vascularity, stem cells with multidirectional differentiation potentials also hold advantages [30, 32]. Besides, stem cells can not only directly differentiate into meniscus cells but also serve as bioactive factors mediators. Among a variety of sources, mesenchymal stem cells were most explored in treating meniscus injuries for their availability, chemotaxis, and immunomodulatory ability [41–44]. In this review, we summarize and evaluate the works promoting the application of mesenchymal stem cells (MSCs) in repairing meniscus injuries in the last 5 years. We also highlight the current challenges and unsolved problems before their successful clinical translation. Finally, the prospective and future development of stem cell-based therapy for meniscus injury are also discussed.

## Anatomy and function of the meniscus

**Fig. 1:**
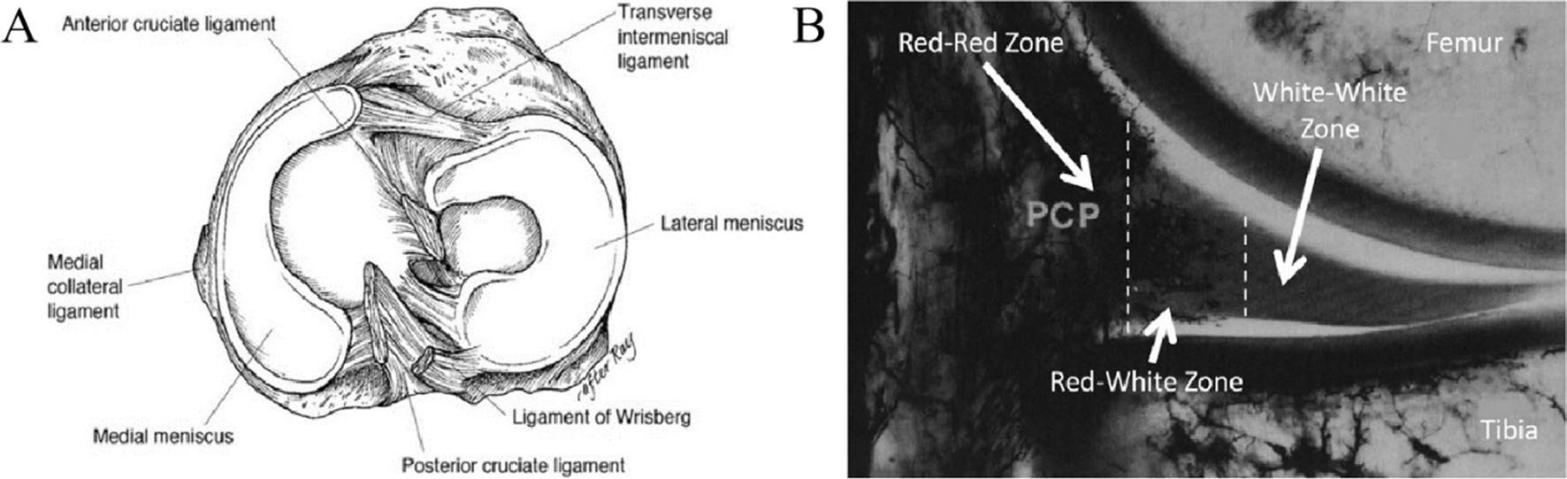
**A** Anatomy of the meniscus viewed from above [66]. Reproduced with permission [66]. Copyright 2002, Wolters Kluwer Health, Inc. **B** Frontal section of the medial compartment of the knee following perfusion with India ink. Vascular red-red zone, avascular white-white zone, and in-between red-white zones are labeled [7]. Reproduced with permission [7]. Copyright 1982, SAGE Publications

The meniscus consists of two crescent-shaped fibrocartilage disks named medial meniscus and lateral meniscus, which are anchored at the anterior and posterior aspects of the tibial plateau. The edge of the meniscus is relatively thick and closely connected with the joint capsule, while the center of the meniscus is thin and in a mobile state [45–47] (Fig. 1A). The joint capsule-derived vessels and nerves infiltrate the peripheral 10–30% of the meniscus, which was called the “red zone” and can be healed after injury [7]. In contrast, the “white zone” refers to the central two-thirds meniscus with no vessels supply or nerves innervation and bears poor intrinsic healing capability [48–50]. The area between the “red zone” and the “white zone” was called the “red-white zone” and displays a transitional self-healing property [7, 50] (Fig. 1B). The cell composition of the meniscus includes fibrochondrocytes and fibroblast-like cells in the peripheral meniscus and chondrocyte-like cells in the middle meniscus [51–54]. There are also amounts of fusiform cells on the superficial layer of the meniscus, which are considered regenerative progenitor cells that play an important role in meniscus repairing [53, 55].

The main components of the meniscus are water, collagen, and proteoglycans [56]. Collagen and proteoglycan endow the meniscus with anti-tensile, compressive, and shear stress functions [57]. The unique biochemical composition and structure dictate the meniscus to convert the vertical load into circumferential hoop stresses and transfer the stresses to the cartilage that possesses a larger surface area, thereby cushioning shock, stabilizing knee joint, and eventually avoiding knee joint injury [58–62]. Furthermore, some studies theorized that the meniscus also accounts for the proprioception and lubrication of the knee joint [63–65].

## Mesenchymal stem cell sources

MSCs derived from bone marrow, synovium, meniscus, and adipose tissue are employed in promoting meniscus regeneration and reconstructing normal meniscus structure [31, 32]. Bone marrow mesenchymal stem cells (BMSCs) are the most frequently used stem cells in regenerative medicine, which can be obtained from the bone marrow of non-weight-bearing bones and possess a strong trilineage differentiation potential [67–70]. Amounts of studies have shown that the transplanted BMSCs can both differentiate into meniscus-like fibrocartilage tissue and enhance the production of extracellular matrix (ECM), thus promoting the integration of regenerated meniscus tissue with the host tissues [71–74]. However, the disadvantages of BMSCs include the painful process of obtaining (bone marrow puncture) and discrepant gene expression profiles with meniscus cells [75]. The number of synovium mesenchymal stem cells (SMSCs) in human synovial fluid was reported to increase after meniscus injury regulated by both calcitonin gene-related peptide and hepatocyte growth factor, indicating SMSCs play an important role in repairing meniscus [76]. Although less in quantity, SMSCs possess a potent chondrogenic potential and have an equivalent or better efficacy in repairing meniscus compared with BMSCs [77–81]. Notably, hierarchical clustering analysis showed that the gene expression profile of meniscus cells is more similar to SMSCs than BMSCs, suggesting a promising perspective of SMSCs in repairing the meniscus [75, 82]. Meniscus-derived mesenchymal stem cells (MMSCs) can be separated from the removed meniscus tissue in arthroscopic surgery or meniscectomy. MMSCs also have trilineage differentiation ability and express stem cell-specific markers [83, 84]. A recent study identified CD146^+^ meniscus cells as the progenitor cells in the meniscus [85]. Gene expression profiling similarity between MMSCs and meniscus-derived chondrocytes is higher than that of MSCs derived from adipose tissue and bone marrow [86]. Some studies also suggest that MMSCs are more inclined to differentiate toward the chondrogenic direction, while BMSCs are more inclined to differentiate toward the osteogenic direction [83]. Adipose-derived stem cells (ADSCs) becomes more and more popular in recent years regarding their accessibility and abundance compared to other MSCs [87]. Although ADSCs were reported to be inferior to BMSCs and SMSCs in terms of chondrogenic and osteogenic differentiation potential [80], amounts of advances have been achieved in both preclinical and clinical studies using ADSCs to treat meniscus injuries.

In summary, MSCs have been widely proved to propose promising therapeutic effects in meniscus repair. The transplanted MSCs can not only directly differentiate into meniscus cells but also can serve as bioactive factors mediators to build a regenerative microenvironment, which significantly facilitates meniscus repair [29, 88]. Each source of MSCs possesses intrinsic merits and demerits and different differentiation potentials and there is no consensus regarding the best source [30]. It’s currently believed that the gene expression profiles of MSCs from intra-articular tissues (such as synovium, meniscus, ligament) are more similar to meniscus cells compared with MSCs from extra-articular tissues (such as muscle, adipose tissue, bone marrow) [86] and are more suitable for meniscus repair.

## Preclinical advances in scaffold-free stem cell therapy

**Fig. 2:**
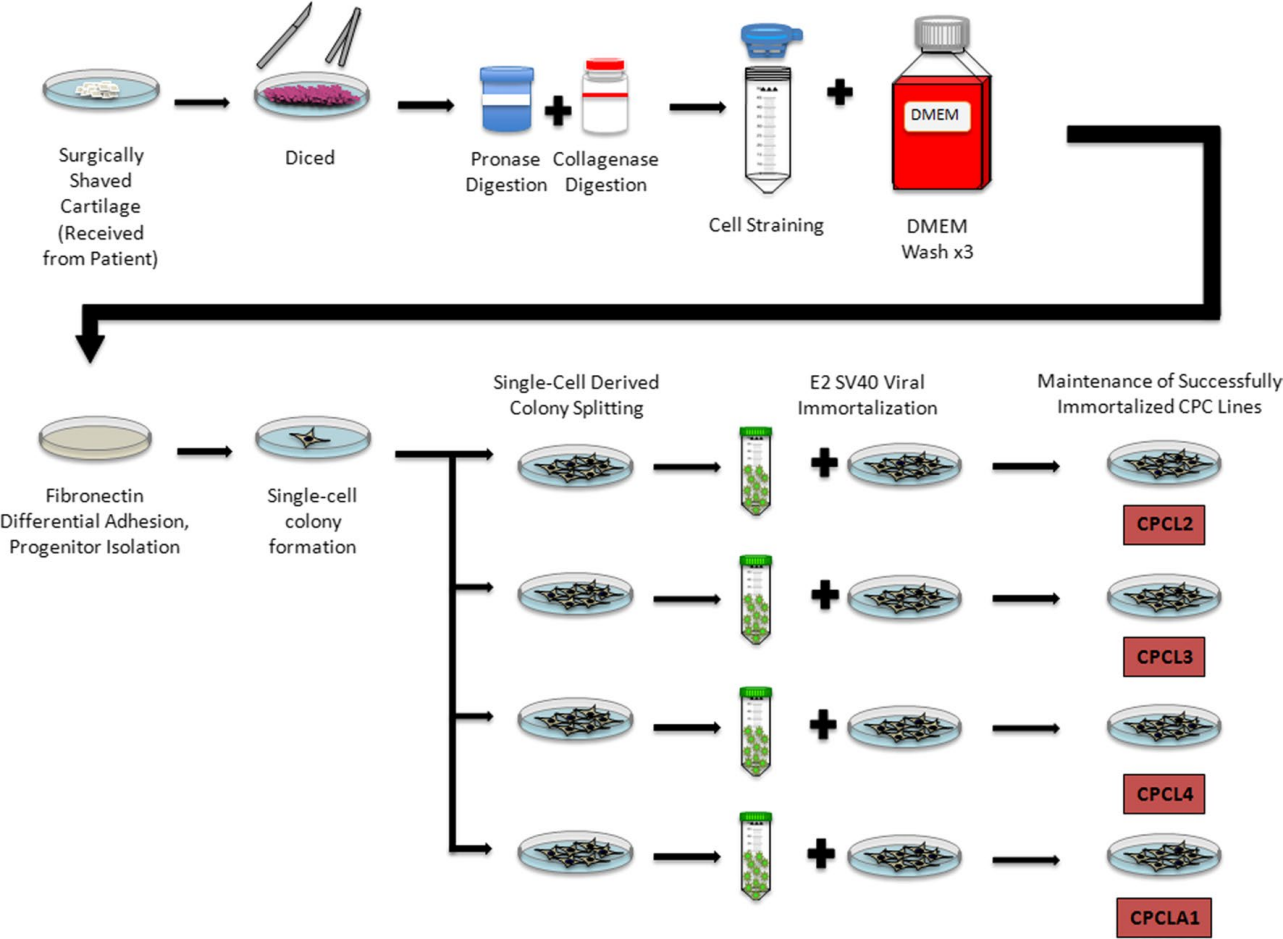
Establishment of healthy human articular cartilage-derived progenitor cell lines [91]. Illustrated diagram of procedure used to establish human cartilage progenitor cell lines. Healthy human cartilage was diced into small pieces and digested using pronase and collagenase. Released cells were washed and strained to remove clumps. Cells were seeded at low density and individual single-cell-derived colonies were separated and stabilized using SV-40 mediated delivery of large-T antigen. Reproduced under the terms of the CC BY-NC license [91]. Copyright 2018, Jayasuriya et al.

Stem cell-based therapies without tissue engineering scaffolds directly implant MSCs to the injured meniscus and have achieved encouraging outcomes in several preclinical studies in the last 5 years (Table 1). The traditional scaffold-free approach often needs to harvest, isolate, and culture MSCs to achieve a suitable number and state of cells before injection. To avoid the complicated procedures, Koch et al*.* [89] invented a one-step stem cell-based therapy, in which bone marrow aspirate concentrate (BMAC) containing BMSCs was harvested and transplanted to the meniscus lesion to induce tissue regeneration in rabbits. Enhanced meniscus tissue regeneration in a time-dependent manner was observed in the BMAC group compared with the platelet-rich plasma (PRP) and control groups. Articular cartilage chondroprogenitor cells (C-PCs) have recently been identified as a favorable stem cell source for meniscus repair for their potent chondrogenic potential [90–92]. For example, Jayasuriya et al*.* [91] isolated C-PCs from healthy human cartilage and investigated their trilineage differentiation capability and meniscus tear repairing potential (Fig. 2). It tuned out the C-PCs express specific mesenchymal stem cell markers and tend to differentiate toward chondrogenic linage rather than adipogenic and osteogenic linage. The C-PCs possess similar chemotaxis with BMSCs and can migrate to the torn area of the meniscus under the stimulation of stromal cell-derived factor-1 (SDF-1), which was proved to be mediated by the SDF-1/CXCR4 pathway. Apart from chondrogenic tendency and spatial chemotaxis, C-PCs express reduced cellular hypertrophy marker collagen X compared with BMSC, representing a more suitable cell source in repairing meniscus tear. ADSCs are another MSCs that attracting more and more attention in meniscus repair for their easy availability. In a preclinical study conducted by Toratani et al. [93], a 3D scaffold-free allogenic ADSCs were implanted into a 1.5-mm defect in the white area of the meniscus. Favorable cell proliferation and adhesion as well as enhanced histological meniscus healing were observed in partial meniscectomy rabbits. Ozeki et al. [94] built a novel meniscus injury model in minipig (the posterior medial meniscus of minipigs was punctuated 200 times using a 23G needle) and evaluated the therapeutic effects of SMSCs transplantation. Histological results showed the proteoglycan content was significantly increased in the SMSCs group compared with the control group 8 weeks after treatment. A closer T2 value with native meniscus tissue was also observed in the MRI images of the SMSCs group. Apart from MSCs source, animal species is another important factor that decides the anatomy properties and biological responses for transplanted stem cells, so employing primates in experiments before clinical translation is of great value. Thus, Kondo et al*.* [95] conducted a preclinical study involving primates, in which aggregates of autologous SMSCs were transplanted to investigate whether the cells can promote meniscus regeneration in aged cynomolgus. After 16 weeks of treatment, the defected meniscus was proved to recover better in the cell-transplanted group, as investigated by histological and MRI T1rho images, demonstrating the promising value of SMSCs in human meniscus repair.

**Table 1:** Preclinical advances in scaffold-free stem cell therapy in the last 5 years

Injury model	Animal	Cell source	Cell density	Implant form	Stimuli	Duration	Year/References
4 mm longitudinal meniscus tear	Rabbit	BMSCs	–	Bone marrow aspirate concentrate	–	12 weeks	2019/[89]
Meniscus tears	Rat meniscus explant	C-PCs	1.0 × 10^5^	Co-culture	Stromal cell-derived factor-1 (SDF-1)	20 days	2018/[91]
Partial meniscectomy	Rabbit	ADSCs	–	High-density mesenchymal stem cell, scaffold-free allograft constructs	–	12 weeks	2016/[93]
Punctuated injury	Minipig	SMSCs	2.0 × 10^7^	Suspension	–	8 weeks	2021/[94]
Partial meniscectomy	Cynomolgus	SMSCs	2.5 × 10^5^	Aggregates	–	16 weeks	2017/[95]

## Preclinical advances in scaffold-based stem cell therapy

The advances in tissue engineering that involved amounts of biomaterials-based natural and synthetic scaffolds have significantly contributed to meniscus repair [96–98]. Scaffolds were designed to possess different compositions and microstructures to induce the adhesion, proliferation, and directional differentiation of seeded MCSs. Bioactive factors with potent biological activity are also employed to promote the biological responsive and regenerative properties of the seeded MCSs. During the last 5 years, MSCs derived from bone marrow, synovium, adipose, and tonsil are widely explored with the combination of tissue engineering scaffolds for meniscus repair (Table 2).

**Table 2:** Preclinical advances in scaffold-based stem cell therapy in the last 5 years

Injury model	Animal	Cell source	Cell density	Scaffold	Stimuli	Duration	Year/References
Punch defect in the lateral meniscus with early osteoarthritis	Rabbit	BMSCs and meniscal cells	1.0 × 10^6^	Collagen–hyaluronan	–	12 weeks	2017/[99]
Full-thickness defect	Rat	BMSCs	5.0 × 10^5^	Decellularized meniscus extracellular matrix	–	2 months	2020/[100]
Full-thickness meniscus defect	Rat	BMSCs	7.5 × 10^5^	Decellularized meniscus extracellular matrix	–	4 weeks	2018/[101]
Meniscectomy	Rabbit	BMSCs	5.0 × 10^6^	3D-printed Poly(e-caprolactone) (PCL)	–	24 weeks	2017/[104]
Punch defects	Rabbit	BMSCs	4.0 × 10^6^	Thermosensitive, injectable, in situ crosslinked hydrogel	TGF-β1	8 weeks	2020/[105]
Punch defects	Rabbit	BMSCs	1.0 × 10^6^	Kartogenin-platelet-rich plasma gel	Kartogenin and platelet	3 months	2019/[106]
Radial defects (5 mm width)	Rabbit	SMSCs	1.6 × 10^6^	Aligned electrospun nanofibrous	–	12 weeks	2018/[108]
Punch defects	Rabbit	ADSCs	4.0 × 10^5^	Polycaprolactone/silk/fibroin/gelatin/ascorbic acid composite	–	2 months	2021/[110]
Full-thickness radial tear spanning 90% of the medial meniscal width	Goat	ADSCs	2.0 × 10^6^	Methacrylated gelatin hydrogel	TGF-β3	6 months	2019/[112]
Unilateral total medial meniscectomy	Rabbit	ADSCs and chondrocytes	2 × 10^4^	Polyvinyl alcohol/Chitosan	–	7 months	2017/[113]
Full-thickness cylindrical defects (diameter = 1.5 mm)	Rabbit	Tonsil-derived mesenchymal stem cells	1.5 × 10^5^	Riboflavin-induced photocrosslinked collagen–hyaluronic acid hydrogel	TGF-β1	10 weeks	2017/[117]
Meniscectomy	Rat	Tendon-derived stem cells and SMSCs	–	Decellularized semitendinosus tendon	–	24 weeks	2017/[118]
Avascular meniscal tear	Sheep	BMSCs	3.0 × 10^5^	Collagen	–	13 weeks	2017/[119]

### Bone marrow mesenchymal stem cells (BMSCs)

**Fig. 3:**
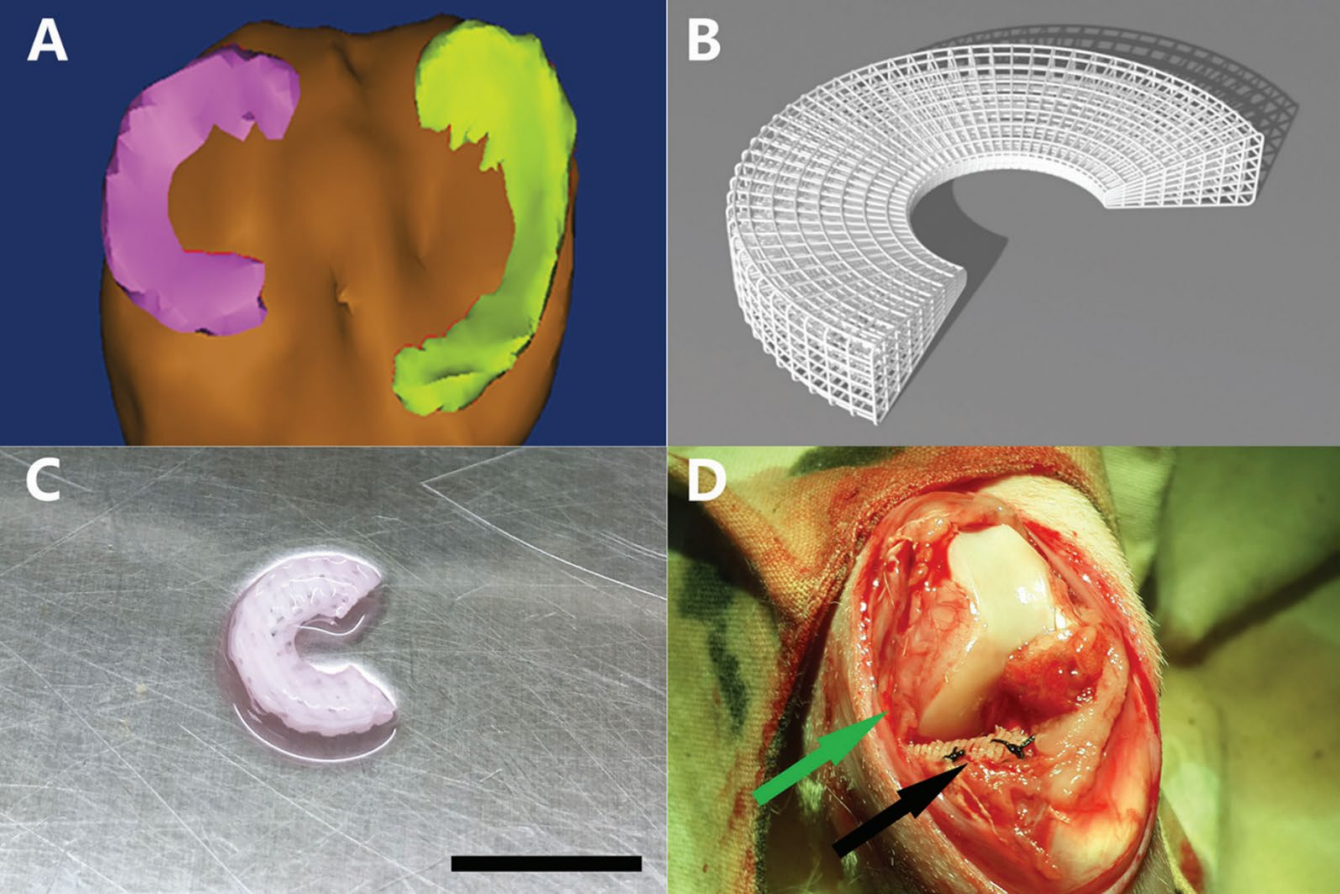
**A** Anatomical reconstruction model of rabbit menisci in left knee [104]. **B** A typical model of 3D medial meniscal scaffold. **C** 3D-printed poly(e-caprolactone) (PCL) scaffold seeded with mesenchymal stem cells (scale bar represents 10 mm). **D** PCL scaffold (black arrow) implanted between femur and tibia, with medial collateral ligament (green arrow) reserved. Reproduced with permission [104]. Copyright 2017, SAGE Publications

BMSCs are still the most popular MSCs for meniscus repair in the last 5 years. Compared with meniscus cells, BMSCs show comparable therapeutic effects for meniscus defect but express significantly more collagen II gene in a situation of early osteoarthritis [99]. Scaffolds made of natural-derived materials hold the advantages of good biocompatibility and were used to load and deliver BMSCs to repair the meniscus, of which decellularized meniscus matrix scaffold attract a lot of attention for it can inhibit the hypertrophic differentiation of seeded BMSCs and enhance the meniscus extracellular matrix production. Zhong et al*.* [100] investigated the meniscus repair performance of an injectable BMSCs-encapsulated decellularized meniscus extracellular matrix scaffold in rats. Histological and micro-CT results demonstrated the decellularized meniscus extracellular matrix scaffold can significantly promote the BMSCs fibrochondrogenic markers expression and prevent the osteoarthritis development compared with collagen hydrogel scaffold. Furthermore, BMSCs cultured in decellularized meniscus extracellular matrix scaffold produced more collagen I, collagen II, and aggrecan, presenting a closer phenotype to meniscus cells. In another study, an injectable biomimetic scaffold was designed using decellularized meniscus ECM hydrogel to deliver BMSCs for meniscus repair. The BMSCs were retained 8 weeks in the scaffold after implantation, which greatly contribute to the integrative repair of a full-thickness meniscus defect in rats [101]. Apart from decellularized meniscus matrix scaffolds, other natural source-based scaffolds have also been explored. Ying et al*.* [102] designed and fabricated a porous silk fibroin scaffold and used it to load BMSCs to repair meniscus injury in rabbits. The porous structure of the scaffold benefits BMSCs adhesion and proliferation as well as facilitates meniscus regeneration. 6 and 12 weeks after implantation, significant positive glycosaminoglycan (GAG), collagen I, collagen II, and S100 protein staining were observed around arranged fibrous cartilage-like neo-tissue in the defect area in the experimental group. Hu et al*.* [103] fabricated a novel sliver nanoparticle using *Bauhinia acuminate* plant flower extract. The silver nanoparticle can promote the proliferation and osteogenic differentiation of BMSCs as well as accelerate the healing process of the meniscus, which was attributed to its outstanding anti-inflammatory, anti-microbial, and cell chemotaxis properties. In addition to nature-derived materials, the synthetic material-based scaffolds were also used as a stem cell loading and delivery system for meniscus repair. Zhang et al*.* [104] fabricated a novel 3D-printed poly(e-caprolactone) scaffold augmented with BMSCs. Compared with cell-free scaffold, the BMSCs-seeded scaffold can significantly promote the regeneration of the meniscus-like tissue and prevent the degeneration of articular cartilage (Fig. 3). Chen et al*.* [105] designed a thermosensitive, injectable, in situ crosslinked hydrogel with good biocompatibility and sustained release ability. The hydrogel was proved to promote the proliferation and fibrochondrogenic differentiation of BMSCs with the assistance of transforming growth factor-β1 (TGF-β1). Eight weeks after transplantation, amounts of fibrocartilaginous tissue expressing strong type II collagen intermingled with weak type I collagen were observed in the defect areas of the meniscus in the BMSCs-TGF-β1-hydrogel group, proposing an alternative therapy for meniscal injuries. Some studies pretreated the BMSCs with drugs or bioactive factors to further promote their regenerative ability and therapeutic effects. In a study designed by Liu et al*.* [106], the isolated BMSCs were cultured with various concentrations of kartogenin (KGN) for 2 weeks before transplantation. In vitro studies showed the chondrogenesis property of KGN co-cultured BMSCs increased in a KGN concentration-dependent manner. After implantation, BMSCs-containing gel showed better regenerative effects than BMSCs-free gel, which can be further enhanced by the addition of KGN and realize complete meniscus healing. Despite the advances in scaffold-based therapy, the mismatch between transplanted implants and patient-specific lesion shape still limits their application. To address the issue, a patient-specific BMSCs-loaded 3D bioprinting meniscus scaffold was designed and fabricated by Filardo et al*.* [107], which can fabricate perfect anatomical-match constructs with the native meniscus of patients by collecting and processing Digital Imaging and Communications in Medicine data from MRI scans.

### Synovium mesenchymal stem cells (SMSCs)

**Fig. 4:**
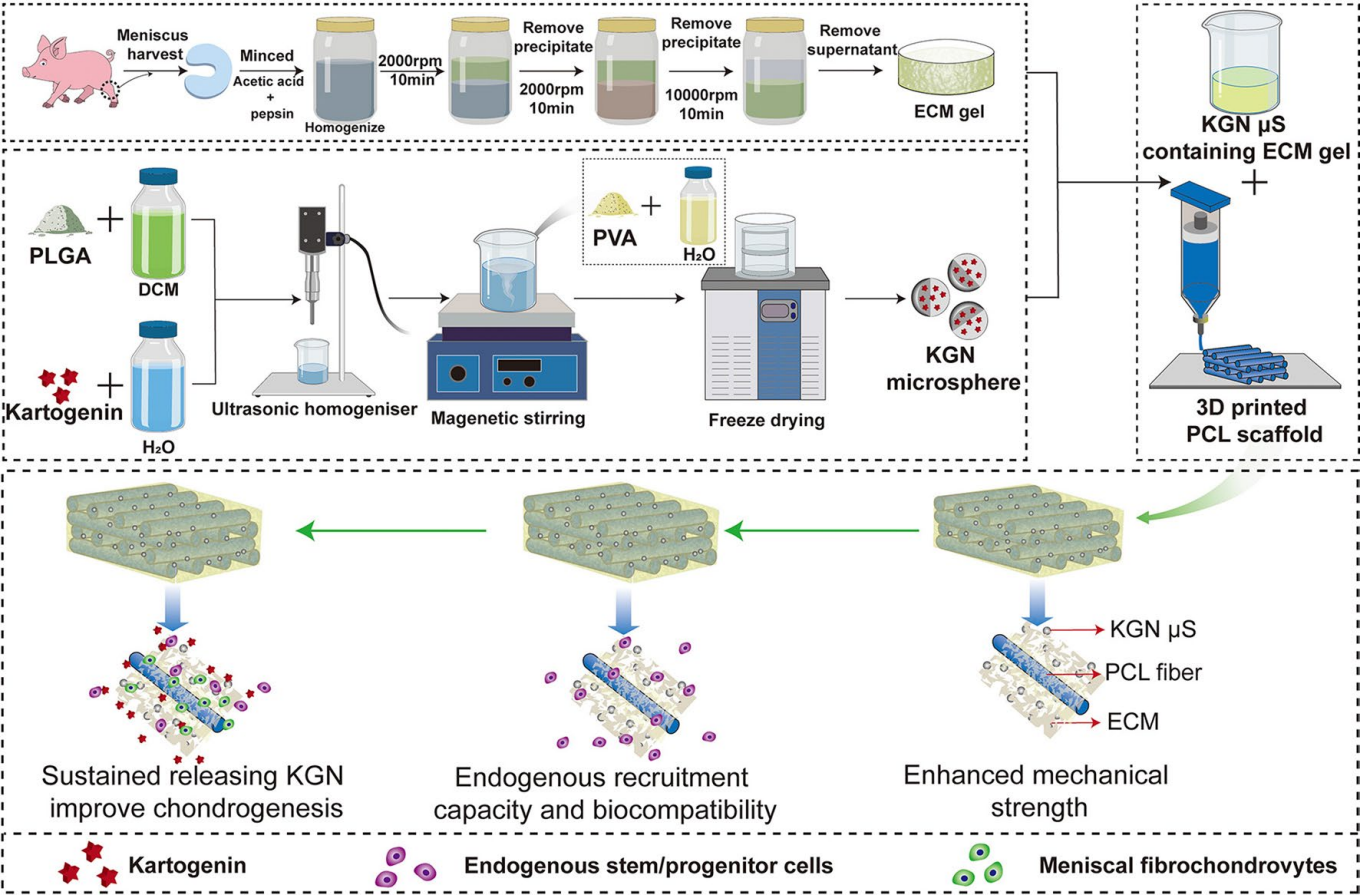
Schematic representation of the preparation process of the scaffolds [109]. Flow chart of the preparation of the **A** MECM gel, **B** KGN-containing PLGA microspheres, and **C** PCL/MECM-KGN µS scaffold; **D** Possible mechanism of meniscus regeneration. Enhanced mechanical strength, endogenous stem cells, and sustained releasing KGN contributed to meniscus regeneration in these experiments. Reproduced under the terms of the CC BY-NC license [109]. Copyright 2021, Li et al*.* MECM, meniscus extracellular matrix; KGN, kartogenin; PLGA, poly(lactic-co-glycolic) acid; µS, microspheres

Synovial tissue was considered a favorable MSCs source for its potent osteogenic, chondrogenic, and adipogenic capacities. In meniscus repair, SMSCs were considered to possess comparable or better therapeutic effects than BMSCs [77–81]. Recently, Shimomura et al*.* [108] designed an aligned electrospun nanofibrous scaffold with fiber direction matching that of the meniscal circumferential fibers. The scaffold was combined with SMSCs to repair damaged meniscus in a rabbit model. With favorable structural similarity and regenerative properties, the therapy can significantly promote new fibrocartilaginous tissue formation as well as prevent meniscal extrusion and articular cartilage degeneration. In another study, Li et al*.* [109] fabricated an ingenious scaffold as a drug and stem cell delivery system, which composed of 3D-printed PCL, meniscus extracellular matrix (MECM), and KGN-loaded poly(lactic-co-glycolic) acid (PLGA) microsphere (Fig. 4). The MECM and released KGN from the scaffold were proved to promote the adhesion, proliferation, and chondrogenic differentiation of the co-cultured SMSCs. Furthermore, SMSCs seeded in the scaffold presented a synergistic therapeutic effect with sustained released KGN in promoting the biocompatibility and chondrogenic properties of the scaffold.

### Adipose-derived stem cells (ADSCs)

ADSCs attract much attention in regenerative medicine for their easy accessibility and high yield. Concerning meniscus repair, ADSCs can promote the regenerative properties and biological responses of co-transplanted scaffolds. For example, Abpeikar et al*.* [110] designed a novel scaffold composed of polycaprolactone/silk fibroin/gelatin/ascorbic acid. The scaffold possesses suitable mechanical properties and was seeded with ADSCs to repair the meniscus. The new meniscus regenerated in the scaffold group was proved to exhibit similar histological configuration and ECM deposition with native tissue. Romanazzo et al*.* [111] functionalized an alginate hydrogel scaffold with ECM from the inner and outer regions of the meniscus and ADSCs. The inner meniscus ECM can promote the chondrogenesis of the seeded stem cells while the outer meniscus ECM directs the stem cells to differentiate into fibroblastic phenotype. After supplementing transforming growth factor-β3 (TGFβ3), the inner and outer meniscus ECM can further promote the infrapatellar fat pad-derived stem cell proliferation and differentiation as well as meniscus tissue regeneration. The mechanical properties of the scaffold can be strengthened by the combination of PCL microfibers and present a promising therapy for clinical meniscus injury. In another study, a severe meniscus tear model spanning 90% meniscus width was built in goat by Rothrauff et al*.* [112]. Such serve tear was proved to bear poor healing process and would lead to cartilage degeneration and osteoarthritis after 6 months. A photocrosslinkable hydrogel loading ADSCs and TGF-b3 that procured intraoperatively was designed and implanted to augment the healing process of the meniscus tear. Compared with the suture-only group, the ADSCs-seeded hydrogel group showed significantly enhanced neo-tissue regeneration and chondroprotection abilities. However, Moradi et al*.* [113] demonstrated ADSC has no significant contribution to the healing process of meniscus injuries. They found the macroscopic, histological, and immunofluorescent outcomes of the articular chondrocytes-ADSCs scaffold are inferior to the articular chondrocytes scaffold. The chondroprotective property of the articular chondrocytes-ADSCs scaffold was even worse than the cell-free scaffold. It’s hypothesized that ADSCs had undergone morphological changes from fibroblast to sphere to present a mature hyaline phenotype, which significantly limited their chondrogenic property. So, further studies are needed to identify the therapeutic effects of ADSCs for meniscus repair.

### Tonsil-derived mesenchymal stem cells (T-MSCs)

Tonsil-derived mesenchymal stem cells (T-MSCs) from tonsillectomy hold advantages for their accessible and minimally invasive harvest procedure and have been explored in treating multiple musculoskeletal diseases [114–116]. Koh et al*.* [117] extracted a conditioned medium of meniscal fibrochondrocytes and TGF-β3 to expand T-MSCs. The T-MSCs were then encapsulated in riboflavin-induced photocrosslinked collagen–hyaluronic acid hydrogels to repair meniscus injuries. It turned out that conditioned medium-expanded T-MSCs followed by TGF-β3 exposure can promote fibrocartilage-related genes expression and extracellular matrix components production. Enhanced cell proliferation, glycosaminoglycan accumulation, and collagen deposition were observed in the CM-expanded T-MSCs group, indicating tonsil is a favorable stem cell source for meniscus repair.

### Combined application of different cell sources

The combined application of MSCs from different sources was considered to propose superior effects in repairing the meniscus. Li et al*.* [118] utilized autogenous semitendinosus tendon as scaffold loading tendon-derived stem cells and SMSCs to reconstruct meniscus in rats. In vivo tests demonstrated the scaffold possesses favorable fibrochondrogenic and chondroprotective properties. Regenerated fibrochondrocytes, proteoglycan, and collagen were observed in the defect areas of the meniscus. Notably, the neo-meniscus tissue has similar biomechanical and chondroprotective properties with the native meniscus, indicating a promising application of combined MSCs from different sources.

## Clinical advances in stem cell-based therapy

**Fig. 5:**
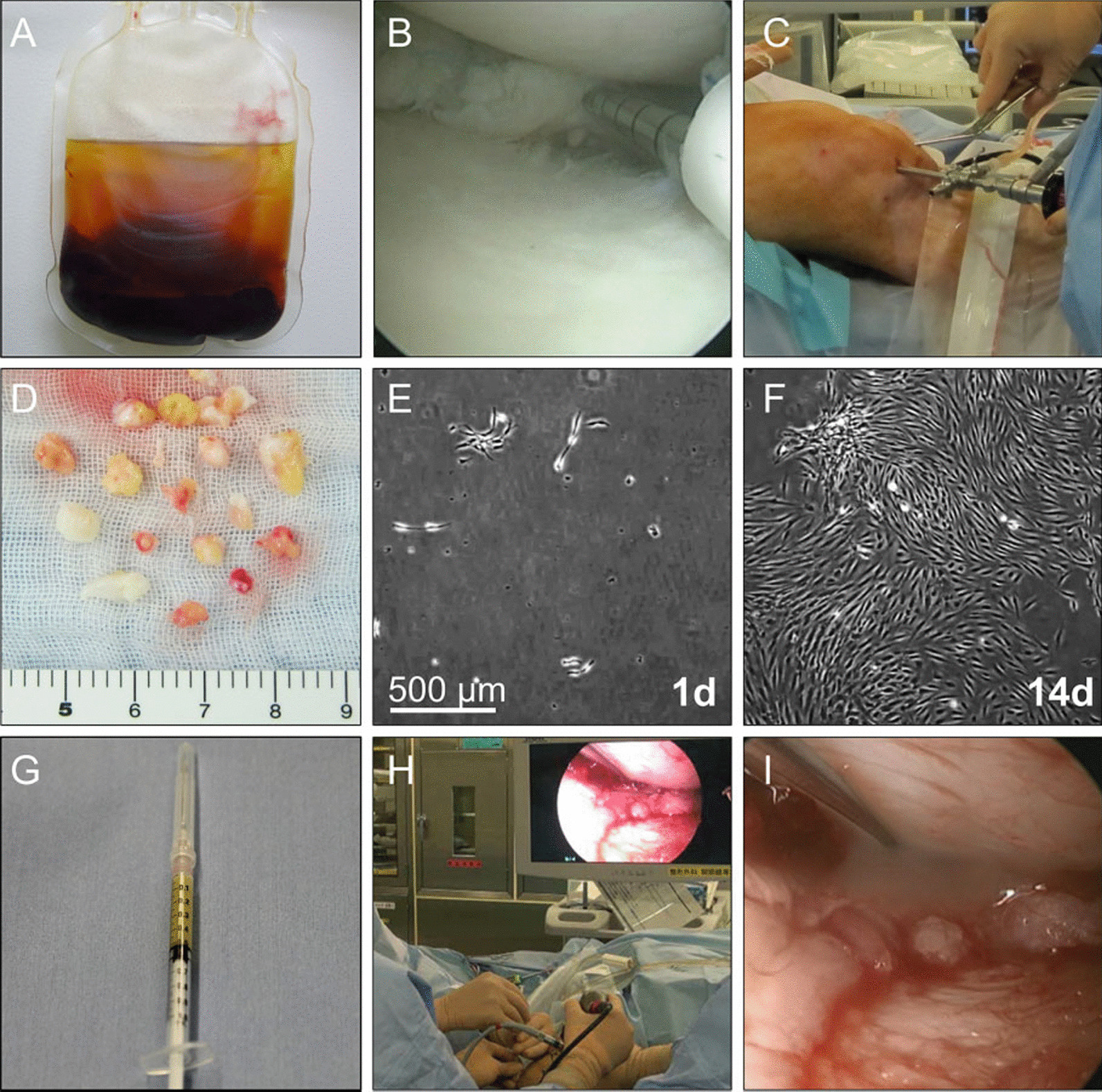
Procedure for transplantation of synovial MSCs onto the repaired meniscus [120]. **A** Whole blood after centrifugation to prepare autologous human serum. **B** Arthroscopic meniscal repair. **C** Synovium harvest with a pituitary rongeur. **D** Synovium tissues as an MSC source. **E** SMSCs 1 day after plating. **F** SMSCs 14 days after plating. **G** SMSC suspension in a syringe. **H** Arthroscopic transplantation of SMSCs. (I) SMSC suspension was placed onto the repaired meniscus. Reproduced under the terms of the CC BY-NC license [120]. Copyright 2019, Sekiya et al.

Many clinical trials have been conducted to evaluate the effects of stem cell therapy in meniscus repair and achieved encouraging outcomes. A first-in-human study that combined surgical repair and SMSCs transplantation was conducted by Sekiya et al*.* [120]. In this clinical trial, 5 patients (mean age of 48.2 years, all-male) suffering from complex degenerative tear of the medial meniscus received surgical meniscus repair and subsequent SMSCs transplantation. Specifically, the meniscus tear was firstly treated by a standard surgical procedure using all-inside and inside-out repair techniques. Then a suspension of in vitro cultured autologous SMSCs was transplanted onto the repaired meniscus using an 18-gauge needle attached to a 1 mL syringe 2 weeks after the surgery (Fig. 5). Two years after SMSCs transplantation, increased scores for “pain,” “daily living,” and “sports activities” were presented in these patients. No adverse event occurred except an increase in c-reactive protein, a joint effusion, and a localized warmth of the knee were recorded, which can both be relative to the meniscal repair surgery or SMSC transplantation. This study suggested the surgical repair in combination with SMSC transplantation has a promising effect in alleviating the clinical symptoms of complex meniscus tear patients. Another clinical trial conducted by Sekiya et al*.* [121] also combined meniscus repair surgery with SMSC transplantation, which treated 6 patients (5 male and 1 female with a mean age of 54 years) with degenerative flaps and radial tears of the medial meniscus using the same treatment procedure. For the 4 flaps tears patients, the tear zone was completely restored to stable and smooth in two patients and partially restored in the other two patients during the 52 weeks follow-up. The other two radial tear patients were also reported to be completely healed at the final follow-up. The arthroscopy score and Lysholm score were significantly higher than the preoperative level in all patients 1 year after being treated by this therapy. Mahajan et al*.* [122] injected the autologous MSCs compound derived from bone marrow and adipose tissue and platelet-rich plasma into the venous and knee joint of a patient (31-year-old female) who suffered from meniscus injury and was unwilling to accept surgery. Improved clinical parameters and enhanced meniscus and ligament tissue regeneration were observed according to MRI images 1 year after treatment, indicating a promising perspective of using autologous MSCs compound to treat meniscus tear. In addition to cell injection, the combined therapy involved tissue engineering scaffold and stem cells has also been explored in the clinic. For example, Whitehouse et al*.* [119] optimized the autologous BMSCs-seeded collagen scaffold in a sheep meniscal cartilage tear model. Confirming encouraging results in sheep, they conduct a single-center, prospective, open-label study translating the therapy to the clinic to treat 5 patients (mean age of 37 years, 4 male and 1 female) with a complex degenerative tear of the medial meniscus. In this study, autologous BMSCs were isolated, cultured, and transplanted to a collagen-based scaffold at a density of 1.0 × 10^6^/cm^2^. Then the BMSCs/collagen scaffold was inserted into the meniscus lesion through the arthroscope and fixed with a vertical mattress suture. Significantly improved clinical symptoms were observed in three patients who have no clinical and radiological evidence of recurrent tear while the other two patients needed to receive subsequent meniscectomy due to retear or nonhealing of the meniscal tear at approximately 15 months after the initial surgery. This study indicated the combination of collagen-based scaffold implantation with BMSC transplantation can alleviate the clinical symptoms of meniscus tear patients but have a relatively high failure rate. In another study, Olivos-Meza et al*.* [123] firstly employed polyurethane meniscal scaffolds loading with BMSCs to improve the prognosis of patients who underwent meniscectomy. Seventeen patients (13 male and 4 female with a mean age of 36 years) who received meniscectomy were enrolled in the trial. Standard anterolateral and anteromedial arthroscopic portals were employed and the polyurethane scaffold was implanted through a 10-mm cannula and sutured to the capsule and native meniscus borders using all-inside, inside-out, and outside-in fixation techniques. The therapeutic effects of transplanting acellular polyurethane scaffold (APS) or polyurethane scaffold enriched with BMSC (MPS) were evaluated and compared by T2 mapping at 12 months postoperatively. The T2 mapping values of the tibia in the MPS group increased slightly at 9 months and returned to initial values at 12 months, while a significant decline from 3 to 12 months was observed in the APS group. At the final time point, the difference tended to be negligible between the MPS group and the APS group (*P* > 0.05), suggesting no benefit was obtained by the addition of BMSCs. Thus, further studies involving more patients and longer follow-up times are needed to evaluate the effects of MSCs as adjuvant therapy for the polyurethane scaffold implantation (Table 3).

**Table 3:** Clinical advances in stem cell-based therapy in the last 5 years

Clinical indication	No. of patients	Mean age	Male:Female	BMI (kg/m^2^)	Cell source	Cell count	Administration route	Stimuli	Follow-up	Clinical outcome	Level of evidence	Year/References
Avascular meniscal tear	5	37 yr	4:1	25 (median)	BMSCs	1.0 × 10^6^/cm^2^	collagen scaffold	FGF-2	2 years	IKDC, Tegner–Lysholm score, ROM	4	2107/[119]
Complex degenerative tears of the medial meniscus	5	48.2 yr	All male	25.9 (mean)	SMSCs	3.2–7.0 × 10^7^	Intra-articular injection	–	2 years	Lysholm, KOOS, NRS	4	2019/[120]
Medial meniscus tear	6	54 yr	5:1	23 (mean)	SMSCs	4.0 × 10^7^	Intra-articular injection	–	1 year	Lysholm	4	2021/[121]
Medial and lateral meniscal and anterior cruciate ligament tears	1	31 yr	Female	/	BMSCs and ADSCs	500–5000 × 10^6^ BMSCs, 1600–400 × 10^6^ ADSCs	Intra-articular and intravenous injection	Platelet-rich plasma	1 year	/	5	2021/[122]
Meniscectomy history	17	36 yr	13:4	27.1 (mean)	Peripheral blood MSCs	2.0 × 10^7^	Polyurethane meniscal scaffolds	–	1 year	Lysholm	3	2019/[123]

BMI, Body mass index; FGF-2, Fibroblast growth factor-2; IKDC, The International Knee Documentation Committee score; ROM, Range of motion; KOOS, Knee Injury and Osteoarthritis Outcome Scale score; NRS, Numerical Rating Scale

In conclusion, the clinical trials published during the last 5 years demonstrated encouraging results using stem cell-based strategies to repair meniscus tears. Most treated patients obtained knee joint function improvement and pain relief. However, the small number of patients, absence of a control group, short follow-up time greatly limit the credibility of these studies. Three studies combine the standard surgical treatment with intra-articular injection of stem cells, which bear a serious risk of bias for the absence of a control group (isolated standard surgical treatment). Furthermore, the only study that set up a control group conducted by Meza et al*.* demonstrated no benefit was obtained by the addition of BMSCs compared with acellular polyurethane scaffold. The considerable heterogeneity of these studies also makes it difficult to conclude the therapeutic effects of stem cell-based strategy for meniscus regeneration. Thus, large multi-center clinical projects are needed to further validate the full utility of these stem cell-based therapies for meniscus injury.

## Discussion

According to the available literature, several elements should be considered to promote the therapeutic effects and translational prospect of stem cell-based therapy for meniscus repair. Firstly, the safety of the transplanted stem cells should be emphasized. The gene expression profile of long-time in vitro culture autologous stem cells will change, so the possibility of carcinogenesis and damaged differentiation potential should be paid attention. Also, it should be alert to autoimmune reactions if the allogeneic stem cells are involved for it can lead to serious clinical consequences [124]. Secondly, concerning the medium of culturing MSCs for meniscus repair, the serum-free medium containing TGF-β and dexamethasone that can promote proteoglycans production and integrative repair was considered the priority for MSC-seeded scaffolds, while the serum-containing medium is more suitable for the meniscus tissue composition-based scaffolds [91]. Thirdly, although stem cells as a growth factor mediator can produce and regulate the expression and activity of many bioactive factors, the involvement of bioactive factors that can induce cell differentiation and meniscus regeneration may help enhance the therapeutic effects of cell-based therapy. For example, the meniscus-derived decellularized matrix was reported to need the assistance of transforming growth factor beta-3 (TGF-β3) and insulin-like growth factor-1 (IGF-1) to direct the chondrogenic differentiation of synovial fluid-derived mesenchymal stem cells [125]. Tarafder et al*.* [126, 127] evaluated the effects of connective tissue growth factor and TGF-β3 in repairing avascular meniscus tears. The short-term high dose release of connective tissue growth factor was proved to recruit stem cells to tear site and produce integrated fibrous matrix while sustained slow release of TGF-β3 can induce the fibrous matrix to convert into the fibrocartilaginous matrix, thus achieving seamless healing of avascular meniscus tears. Apart from transforming growth factors β1 and β3, Mohawk is another key transcription factor identified recently that can promote meniscus cell phenotype and tissue repair [128]. RNA sequencing data from 37 human tissues in the Genotype-Tissue Expression database and meniscus and articular cartilage showed Mohawk factor is highly expressed in the meniscus. The Mohawk transcription factor can also induce BMSCs to differentiate into meniscus cell phenotype with the cooperation of TGF-β3. Adenoviral-MKX-transduced BMSCs-loaded decellularized meniscus scaffold has achieved encouraging outcomes in repairing meniscus tear with increased glycosaminoglycan content, extracellular matrix interconnectivity, and biomechanical properties. Besides, SDF-1 was also hypothesized as a factor that can stimulate MSCs to differentiate to meniscus cell phenotype. Intra-articular administration of SDF-1 can recruit macrophages, CD90-positive cells, and CD105-positive cells to the defect area and contribute to meniscus healing [129]. SDF-1 preconditioned C-PCs can successfully migrate from the scaffold and adhere to meniscus lesions to stimulate bridging of meniscus tears. However, the SDF-1 preconditioned C-PCs didn’t express more chondrogenic genes [130]. Thus, further studies are needed to investigate the underlying relationship between SDF-1 and meniscus regeneration. Fourthly, complementary clinical techniques, including novel stem cell isolation and transplantation method and the corresponding postoperative radiographical evaluation, should receive more attention and be innovated to promote the clinical transformation of stem cell therapy. An example is the sonographically guided knee meniscus injection technique invented by Baria et al*.* [94], which provides an opportunity to deliver stem cells to the specific injury area of the meniscus accurately and safely.

Despite the fact that many advances have been achieved in stem cell-based therapy, obstacles remain before their successful clinical translation. Firstly, the studies focusing on applying stem cells in meniscus repair are heterogeneous in animal models, cell sources, and scaffolds, and too limited comparative studies are available to conclude the most promising stem cell [29]. Martin et al*.* [131] once conducted a comparative study evaluating the regenerative capacity of articular chondrocytes, meniscus cells, fat pad cells, and synovial membrane cells in a hyaluronan-based scaffold for meniscus repair to identify the best cell source that available from knee joint arthroscopy. It tuned out only articular chondrocytes possess the ability to form neo-meniscus that contains relevant amounts of collagen and glycosaminoglycan and presents compatible cell phenotypes with the inner and outer native meniscus regions. However, stem cell-related data regarding the best cell source is not available and further comparative researches are needed. Secondly, although many studies suggested that higher stem cell number is related to better therapeutic effects in repairing meniscus, there is currently no consensus on the relationship between cell number and repair effects [89, 90]. A study demonstrated the effect of producing anti-inflammatory cytokines by MSCs after injuries were time and cell number dependent and 6 weeks and 10^6^ cells were considered the lower limit [132–135], while another study proved the largest amount of BMSCs did not correspond to the best quality and largest quantity of bridging tissue in repairing meniscus [136]. However, with the same cell number, transplantation of aggregates of MSCs was considered to have better therapeutic effects than suspension of MSCs [78]. Due to the strict monitoring of the clinical application of stem cell therapy, a low limit cell number with effective therapeutic effect needed to be determined in further studies. From another perspective, the modification and conditioned culture that can increase the chondrogenic-differentiation potential and activity of the isolated stem cells may be a promising solution for this problem. For example, the culture medium containing TGF-β is considered to endow the stem cells with extra meniscus-like tissue regeneration ability and promote proteoglycans production and integrative repair after transplantation [91]. Besides, cell-free strategies that focus on recruiting endogenous stem cells to the injured areas of the meniscus can also be a solution and are attracting increasing attention in recent years [137, 138]. For example, Ruprecht et al*.* [139] demonstrated the meniscus-derived matrix scaffolds can promote meniscus repair by recruiting endogenous meniscus cells from the surrounding microenvironment, both endogenous meniscal cells and exogenously seeded BMSCs can infiltrate the meniscus-derived matrix scaffolds and show comparable ability in promoting the integrative repair of a meniscal tear. Thirdly, a well-designed scaffold can not only provide a 3D support but also a suitable physicochemical environment that can keep the self-renewal and induce directional differentiation of loaded stem cells [37]. Scaffold-based 3D assemblies of stem cells also enhance intercellular interaction, which is essential to promote an orchestral tissue regeneration similar to which occurs during embryogenesis [140]. Thus, more ingenious scaffolds are needed for better therapeutic effects of stem cell-based therapy. Constructing a mimicking 3D microstructure is the key point to induce specific tissue regeneration [141]. For example, scaffolds with a porous structure are more likely to possess osteogenesis properties. It’s encouraged to explore a kind of scaffold microstructure that can induce stem cells to differentiate toward meniscus-like fibrocartilage tissue. Apart from specific microstructure, loading bioactive molecular is another method to enhance the biological responsive properties of scaffold [142]. TGF-β, insulin-like growth factor, and connective tissue growth factor can induce stem cells to differentiate toward chondrogenic direction and show a promising future when co-loaded with stem cells in scaffolds. Fourthly, despite many studies demonstrating that transplanted stem cells can promote neo-meniscus tissue regeneration, but if the stem cells can form durable regenerative meniscus comparable to the natural meniscus is still to be confirmed [84]. Thus, future studies should pay more attention to the composition and structure similarity between the regenerated meniscus tissue and the original meniscus. Longer postoperative follow-up should also be involved to assess the long-term structural and functional stability of the neo-meniscus tissue.

## Conclusion

In conclusion, the intrinsic advantages of stem cell therapy such as potent regenerative ability, combined with encouraging results obtained in numerous preclinical and several small clinical trials, convinced us it’s a promising option for repairing meniscus injury. Ingenious-designed and manufactured scaffolds, as well as further identified meniscus-regenerative bioactive factors, can also be combined with stem cell therapy to obtain synergies. Meanwhile, attention should also be paid to the obstructs before the successful clinical translation of stem cell therapies, no matter in technique, or regulatory policy, and more efforts are needed to further optimize the self-renewal and directional differentiation properties of transplanted stem cell to accelerate the realization of stem cell-based therapy for the benefits of meniscus injury patients.

## Data Availability

Not applicable.

## Abbreviations

MSCs: Mesenchymal stem cells
BMSCs: Bone marrow mesenchymal stem cells
ECM: Extracellular matrix
SMSCs: Synovium mesenchymal stem cells
MMSCs: Meniscus-derived mesenchymal stem cells
ADSCs: Adipose-derived stem cells
BMAC: Bone marrow aspirate concentrate
PRP: Platelet-rich plasma
C-PCs: Cartilage chondroprogenitor cells
SDF-1: Stromal cell-derived factor-1
KGN: Kartogenin
PCL: Poly(e-caprolactone)
MECM: Meniscus extracellular matrix
PLGA: Poly(lactic-co-glycolic) acid
SDF-1: Stromal cell-derived factor-1
TGF-β1: Transforming growth factor-β1
TGF-β3: Transforming growth factor-β3
T-MSCs: Tonsil-derived mesenchymal stem cells
GAG: Glycosaminoglycan
APS: Acellular polyurethane scaffold
MPS: Polyurethane scaffold enriched with bone marrow mesenchymal stem cells
BMI: Body mass index
FGF-2: Fibroblast growth factor-2
IKDC: The International Knee Documentation Committee score
ROM: Range of motion
KOOS: Knee Injury and Osteoarthritis Outcome Scale score
NRS: Numerical Rating Scale
